# Advancing lung adenocarcinoma prognosis and immunotherapy prediction with a multi‐omics consensus machine learning approach

**DOI:** 10.1111/jcmm.18520

**Published:** 2024-07-03

**Authors:** Haoran Lin, Xiao Zhang, Yanlong Feng, Zetian Gong, Jun Li, Wei Wang, Jun Fan

**Affiliations:** ^1^ Department of Thoracic Surgery The First Affiliated Hospital of Nanjing Medical University Nanjing China

**Keywords:** immunotherapy, lung adenocarcinoma (LUAD), machine learning, multi‐omics consensus clusters (MOCs), overall survival (OS)

## Abstract

Lung adenocarcinoma (LUAD) is a tumour characterized by high tumour heterogeneity. Although there are numerous prognostic and immunotherapeutic options available for LUAD, there is a dearth of precise, individualized treatment plans. We integrated mRNA, lncRNA, microRNA, methylation and mutation data from the TCGA database for LUAD. Utilizing ten clustering algorithms, we identified stable multi‐omics consensus clusters (MOCs). These data were then amalgamated with ten machine learning approaches to develop a robust model capable of reliably identifying patient prognosis and predicting immunotherapy outcomes. Through ten clustering algorithms, two prognostically relevant MOCs were identified, with MOC2 showing more favourable outcomes. We subsequently constructed a MOCs‐associated machine learning model (MOCM) based on eight MOCs‐specific hub genes. Patients characterized by a lower MOCM score exhibited better overall survival and responses to immunotherapy. These findings were consistent across multiple datasets, and compared to many previously published LUAD biomarkers, our MOCM score demonstrated superior predictive performance. Notably, the low MOCM group was more inclined towards ‘hot’ tumours, characterized by higher levels of immune cell infiltration. Intriguingly, a significant positive correlation between GJB3 and the MOCM score (*R* = 0.77, *p* < 0.01) was discovered. Further experiments confirmed that GJB3 significantly enhances LUAD proliferation, invasion and migration, indicating its potential as a key target for LUAD treatment. Our developed MOCM score accurately predicts the prognosis of LUAD patients and identifies potential beneficiaries of immunotherapy, offering broad clinical applicability.

## 1 INTRODUCTION

Lung cancer constitutes the principal cause of cancer‐related mortality globally.^1^ It is estimated that in the United States, lung cancer claims the lives of 135,000 individuals annually, surpassing the combined mortality rates of prostate, breast, brain and colorectal cancers.^2^ Lung cancer is categorized into two major histological subtypes: non‐small cell lung cancer (NSCLC), accounting for approximately 85% of cases and small cell lung cancer (SCLC), constituting about 15%.^3^ NSCLC is the most prevalent and typically the most aggressive form of lung cancer,^4^ encompassing subtypes such as adenocarcinoma (LUAD) and squamous cell carcinoma (LUSC). LUAD, representing half of all NSCLC cases, emerges as the most common subtype within NSCLC.^5^ LUAD, recognized for its complexity, is significantly influenced by a myriad of oncogenes, cytokines and chemokines.^6^, ^7^, ^8^ The complexity of LUAD is amplified by the tumour microenvironment's (TME) heterogeneity, affecting treatment, progression and response to therapies.^9^, ^10^ Understanding the TME's complex interactions is vital for creating targeted and effective treatment strategies for LUAD's multifaceted nature.

With the continuous advancements in current therapeutic techniques, innovative treatments for NSCLC are being explored using cutting‐edge modalities. Despite significant progress in surgical interventions, chemotherapy, radiotherapy and immunotherapy and the development of targeted therapies for common mutation sites, the prognosis for a large fraction of LUAD patients has not substantially improved due to the heterogeneity of tumours and primary or secondary drug resistance.^11^, ^12^ Immunotherapy, a focal point of extensive research efforts, has shown promising results in some patients. However, a considerable proportion of patients do not achieve clinical benefits from this treatment. This can be attributed to the pronounced heterogeneity of LUAD, with each cancer genome revealing thousands of mutations, including copy number alterations (CNAs), gene fusions and single nucleotide variations (SNVs), benefiting only a small segment of LUAD patients.^13^ Research indicates that LUAD tumour cells can create an inflammation‐prone environment within the TME, attracting various immune cells to interact with tumour cells, including tumour‐associated macrophages (TAMs), myeloid‐derived suppressor cells (MDSCs), mast cells and tumour‐associated neutrophils (TANs), ultimately leading to a highly immunosuppressive TME.^14^, ^15^, ^16^ Understanding the molecular subtypes could offer a resolution to these challenges. Given the high costs and potential severe adverse reactions associated with immunotherapy, there is an urgent need to leverage large‐scale multi‐omics data and advanced machine learning algorithms to identify biomarkers that can effectively predict prognosis and guide immunotherapy management for LUAD patients.

In our research, we combined expression data for mRNA, long non‐coding RNA (lncRNA) and microRNA (miRNA), genomic mutations and epigenomic DNA methylation to develop multi‐omics consensus clusters (MOCs) for LUAD. Following this, we utilized MOCs‐specific hub genes to create a MOCs‐associated machine learning model (MOCM), employing a variety of ten machine learning algorithms. Demonstrated through both training and validation cohorts, the MOCM revealed substantial prognostic capabilities and showed strong efficacy in predicting the efficacy of immunotherapy treatments. Our findings provide pivotal insights into refining molecular subtyping for LUAD, significantly advancing the pursuit of precision and personalized therapeutic approaches.

## 2 METHOD

### 2.1 Integrating and standardizing multi‐omics data

A comprehensive multi‐omics dataset on LUAD was procured from The Cancer Genome Atlas (TCGA) (https://portal.gdc.cancer.gov), encompassing transcriptomic profiling, somatic mutations and clinical data. Moreover, DNA methylation and pan‐cancer RNA‐sequencing (RNA‐seq) datasets, along with corresponding clinical annotations, were sourced from UCSC Xena (https://Xenabrowser.net). To corroborate the robustness of our analyses, six LUAD cohorts were retrieved from the Gene Expression Omnibus (GEO): GSE13213^17^ (*n* = 119), GSE26939^18^ (*n* = 115), GSE29016^19^ (*n* = 39), GSE30219^20^ (*n* = 86), GSE31210^21^ (*n* = 227) and GSE42127^22^ (*n* = 134), serving as validation cohorts. Additionally, LUAD cases undergoing immunotherapy, derived from the OAK^23^ trials—landmark randomized controlled trials examining neoadjuvant immunotherapy and chemotherapy—were integrated into our study. All utilized datasets have been summarized in Table S1 and the clinical characteristics of OAK are summarized in Table S8. To ensure data consistency and maintain analytical comparability, transcriptional datasets were normalized to the Transcripts Per Million (TPM) metric. The ‘combat’ algorithm from the ‘sva’ R package^24^ was deployed to adjust for potential batch effects. Furthermore, a uniform logarithmic transformation was applied to all datasets, establishing a standardized approach to data analysis. To further validate the efficacy of batch correction in our dataset, principal component analysis (PCA) was performed on the included data samples.

### 2.2 Optimizing cluster analysis using the MOVICS package

We utilized the ‘getElites’ function within the Multi‐Omics Integration and Visualization in Cancer Subtyping (MOVICS) R package^25^ to filter gene characteristics. For continuous data such as mRNA, lncRNA, miRNA and DNA methylation, we employed the ‘getElites’ function, setting ‘method = mad’ to select the top 1500 genes exhibiting the highest variability. Subsequently, by adjusting ‘method = cox’ and incorporating clinical data from the cases, we identified prognostically significant genes with a *p*‐value of <0.01. For binary gene mutation data, we initially used the ‘oncoPrint’ function from the Maftools package to identify the 5000 genes with the highest mutation rates. This was followed by further refinement using the ‘method = freq’ to isolate the top 5% of genes with the highest mutation frequency. Data aggregated from these five dimensions were then integrated into our study for comprehensive analysis.

During the initial phase of feature selection, an evaluation was conducted to establish the optimal number of clusters for our investigation. By employing the ‘getClustNum’ function within the ‘MOVICS’ R package, we integrated the Clustering Prediction Index (CPI), Gap Statistics and Silhouette Score to infer the quantity of subgroups. Subsequently, cluster analysis was performed using the ‘getMOIC’ function, which incorporates a comprehensive set of ten algorithms (CIMLR, ConsensusClustering, SNF, iClusterBayes, PINSPlus, moCluster, NEMO, IntNMF, COCA and LRA), adhering to the default parameters provided by MOVICS. This approach facilitated the acquisition of distinct clustering outcomes from each algorithm. To enhance the robustness of our clustering delineation, we amalgamated the results using the ‘getConsensusMOIC’ function, predicated on the principles of consensus clustering. With the distance parameter set to ‘Euclidean’ and the linkage parameter to ‘average,’ this methodology culminated in the derivation of the definitive clustering outcome.

### 2.3 GSVA Analysis and subtype stability evaluation

Utilizing Gene Set Variation Analysis (GSVA), we computed enrichment scores for multiple therapy‐related signatures, encompassing the cancer‐immunity cycle,^26^ hallmark gene sets (available at http://www.gsea‐msigdb.org/gsea/index.jsp) and signatures pertinent to targeted and radiation therapy. The gene sets used are available in Table S2–S5. To assess the stability of subtypes, we initially verified the clustering outcomes using subtype‐specific biomarkers in the validation cohort. Subsequently, the consistency of consensus clustering was compared against the concordance with the nearest template prediction (NTP) and partitioning around medoids (PAM) classifiers.

### 2.4 Developing MOCM through advanced machine learning methodologies

Univariate Cox regression analysis was employed to assess the impact of MOCs‐specific hub genes on the survival of LUAD patients. This was followed by a comprehensive evaluation using ten‐fold cross‐validation, incorporating ten machine learning algorithms, namely Stepwise Cox, Lasso, Ridge, Cox Partial Least Squares Regression (plsRcox), CoxBoost, Random Survival Forests (RSF), Generalized Boosted Models (GBM), Elastic Net (Enet), Supervised Principal Component (SuperPC) and Survival Support Vector Machine (survivin‐SVM). The objective of this approach was to identify the most critical prognostic features through the maximization of the Concordance Index (C‐index).

### 2.5 Assessing MOCM's prognostic value and clinical utility

The prognostic value and potential clinical applications of MOCM were assessed by scoring each sample within the training and validation cohorts based on the derived model, subsequently categorizing samples into high and low MOCM groups. The prognostic significance of MOCM was evaluated using Kaplan–Meier survival curves, while its diagnostic accuracy was determined through ROC curve analysis. Moreover, a systematic review was conducted to identify 114 LUAD‐related prognostic features, and scores for each sample were calculated based on published coefficients. The prognostic prediction capability of all features was assessed within each cohort using the C‐index.

### 2.6 Immune infiltration assessment

Immune cell infiltration was evaluated using a suite of seven distinct algorithms—EPIC, TIMMER, CIBERSORT, CIBERSORT‐ABS, MCPCounter, QUANTISEQ and XCELL—to analyse the composition of immune cells. We then observed the expression differences of immunomodulatory genes (Table S6) between high and low MOCM groups, which were presented by heatmap.

### 2.7 Cell line preparation and culture conditions

A549 and H1299 LUAD cell lines were obtained from the Institute of Biochemistry and Cell Biology at the Chinese Academy of Sciences in Shanghai, China. The culture medium, containing either DMEM or RPMI 1640, was supplemented with 10% fetal bovine serum (FBS) and 1% antibiotics (100 U/mL penicillin and 100 mg/mL streptomycin). It was employed for the maintenance of A549 and H1299 cells, respectively.

### 2.8 siRNA mediated transfection protocol

Small interfering RNA (siRNA sequences were listed in Table S7) transfection was carried out with the Lipo2000 reagent (Invitrogen, Shanghai, China), strictly following the prescribed protocols of the manufacturer. Generally, coverslips within six‐well plates were utilized for the deposition of A549 and H1299 cells, and the transfection of plasmid or siRNA was performed on the subsequent day.

### 2.9 Western blot analysis

Cell lysates were prepared in RIPA buffer, with proteins separated via sodium dodecyl sulfate‐polyacrylamide gel electrophoresis (SDS‐PAGE) and subsequently transferred onto nitrocellulose membranes (Sigma‐Aldrich, St. Louis, MO, USA). Protein visualization was achieved through radiographic autoradiography. An anti‐ACTIN antibody served as the internal control. The primary antibody selected was GJB3 (Abcam, ab236620).

### 2.10 Colony formation capacity evaluation

A quantity of five thousand cells was introduced into each well of a 6‐well plate as part of the colony formation experiment, and conventional growth medium was introduced, later substituted after 1 week. Methanol was utilized for a period of 15 min after the colonies had reached maturity within a two‐week span, followed by staining with 0.1% crystal violet (Sigma) for 30 min. Following this procedure, the resultant clones were quantified to determine the colony‐forming capability of the clones.

### 2.11 Ethynyl deoxyuridine (EdU) incorporation for cell proliferation

EdU labeling and staining processes were conducted by utilizing an EdU cell proliferation detection kit obtained from RiboBio, Guangzhou, China. After cells were introduced into 96‐well plates at a concentration of 5 × 10^3^ cells per well, a 50 μM EdU labeling medium was administered 48 h post‐transfection. The cells were subjected to a 2‐h incubation in a controlled setting at 37°C with 5% CO2. Subsequently, a treatment was applied to the cells using 4% paraformaldehyde and 0.5% Triton X‐100 for anti‐EdU working solution staining. Nuclei were labelled through the utilization of diamidino‐2‐phenylindole. The determination of the percentage of EdU‐positive cells was carried out via fluorescence microscopy.

### 2.12 Wound healing

Cells were placed onto a 6‐well plate and cultivated until achieving a confluence range of 90%–100%. Utilizing a delicate pipette tip, cells at confluence were subjected to incision, followed by dual rinses with phosphate‐buffered saline (PBS). Microscopic images of equivalent positions in each well were recorded at 0 and 48 h utilizing a microscope (Olympus, Tokyo, Japan). The measurement of wound closure extent was assessed as a percentage of wound confluence, employing ImageJ software.

### 2.13 Assessing cell invasion and migration capabilities

Invasion and migration assays were executed utilizing the Transwell system by Corning, which featured 24 wells with an 8 mm pore size, situated in New York, NY, USA. In the context of migration assays, a population of 5 × 10^4^ cells post‐transfection were introduced into the upper chambers of the plates, comprising 350 μL of serum‐free medium, while 700 μL of medium enriched with 10% FBS was introduced into the lower chambers. Matrigel invasion assays entailed the application of Transwell membranes pre‐coated with Matrigel (Sigma–Aldrich). Following a 16‐h incubation, the cells residing on the upper surface were eliminated, and those that traversed the membrane to the lower surface underwent staining with methanol and 0.1% crystal violet. Photographic records were captured utilizing an inverted microscope manufactured by Olympus in Tokyo, Japan.

### 2.14 Flow cytometry analysis

Forty‐eight hours post‐transfection, cells treated with trypsin were harvested and then stained using a fluorescein isothiocyanate (FITC)‐conjugated Annexin V and propidium iodide (PI) employing the FITC Annexin V Apoptosis Detection Kit from BD Biosciences, Franklin Lakes, NJ, USA. The identification of live, dead, early apoptotic and late apoptotic cells was conducted using a flow cytometer (FACScan, BD Biosciences) equipped with Cell Quest software by BD Biosciences. The percentage of apoptotic cells was determined by aggregating the ratios of early to late apoptotic cells.

### 2.15 Statistical analysis

Supporting information for this article can be found in Table S1. Statistical analyses for dichotomous group comparisons were conducted, employing unpaired t‐tests for normally distributed variables and Wilcoxon rank‐sum tests for variables deviating from normal distribution. For analyses exceeding two groups, parametric and non‐parametric variables were analysed using one‐way ANOVA and Kruskal‐Wallis tests, respectively. Contingency tables were assessed via chi‐squared tests complemented by Fisher's exact test for bilateral precision. The demarcation of MOCM scores was determined through the ‘surv_cutpoint’ functionality within the ‘Survminer’ R package.

## 3 RESULTS

**FIGURE 1:**
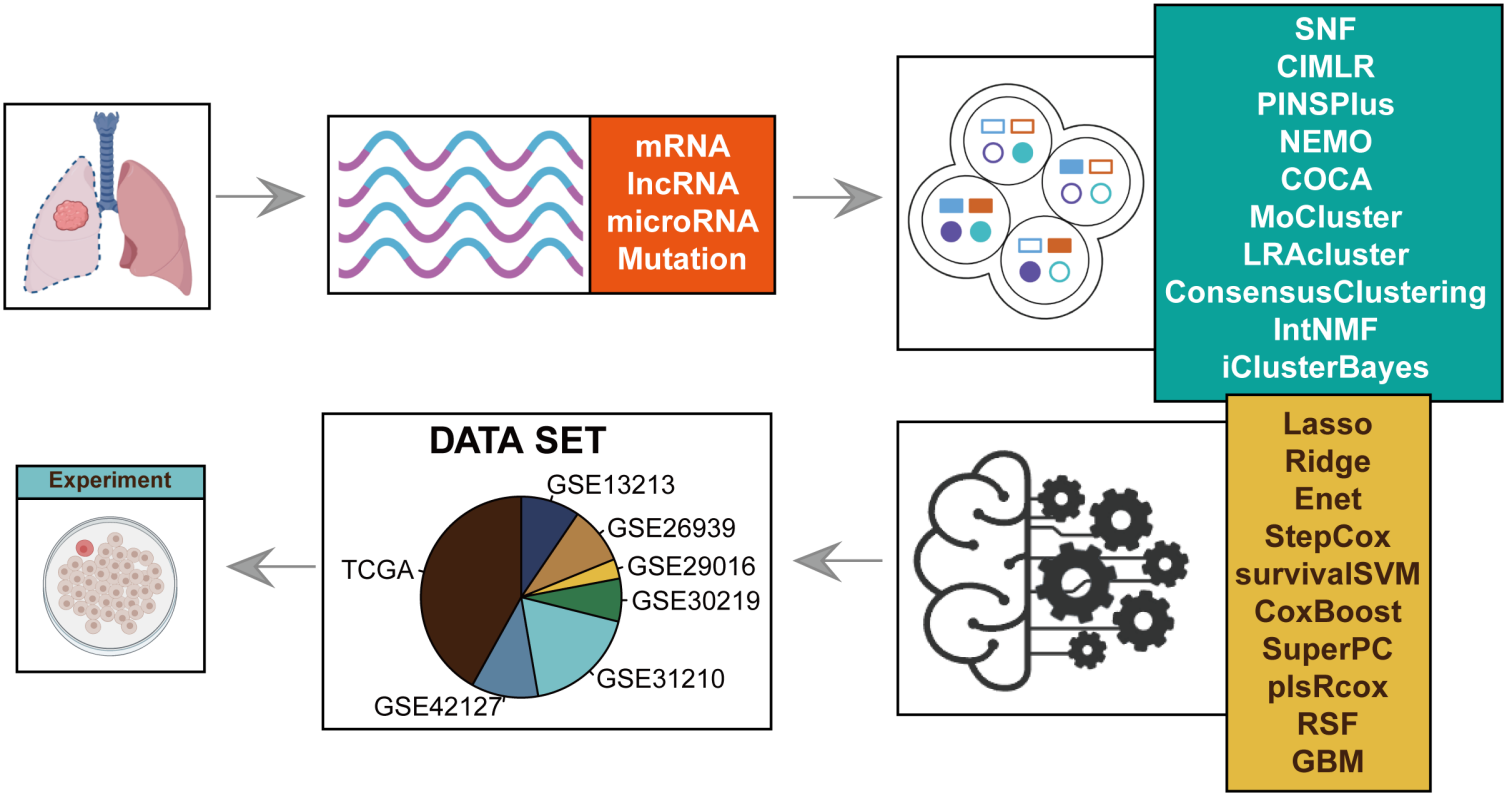
Workflow Schematic. Employing ten clustering algorithms to establish stable multi‐omics consensus clusters (MOCs), followed by the integration of ten machine learning algorithms based on MOC‐specific hub genes to develop a MOCs‐associated machine learning model (MOCM) for prognosis and immunotherapy prediction.

Figure 1 illustrates the primary workflow of this investigation.

### 3.1 Subtype identification and prognostic validation

**FIGURE 2:**
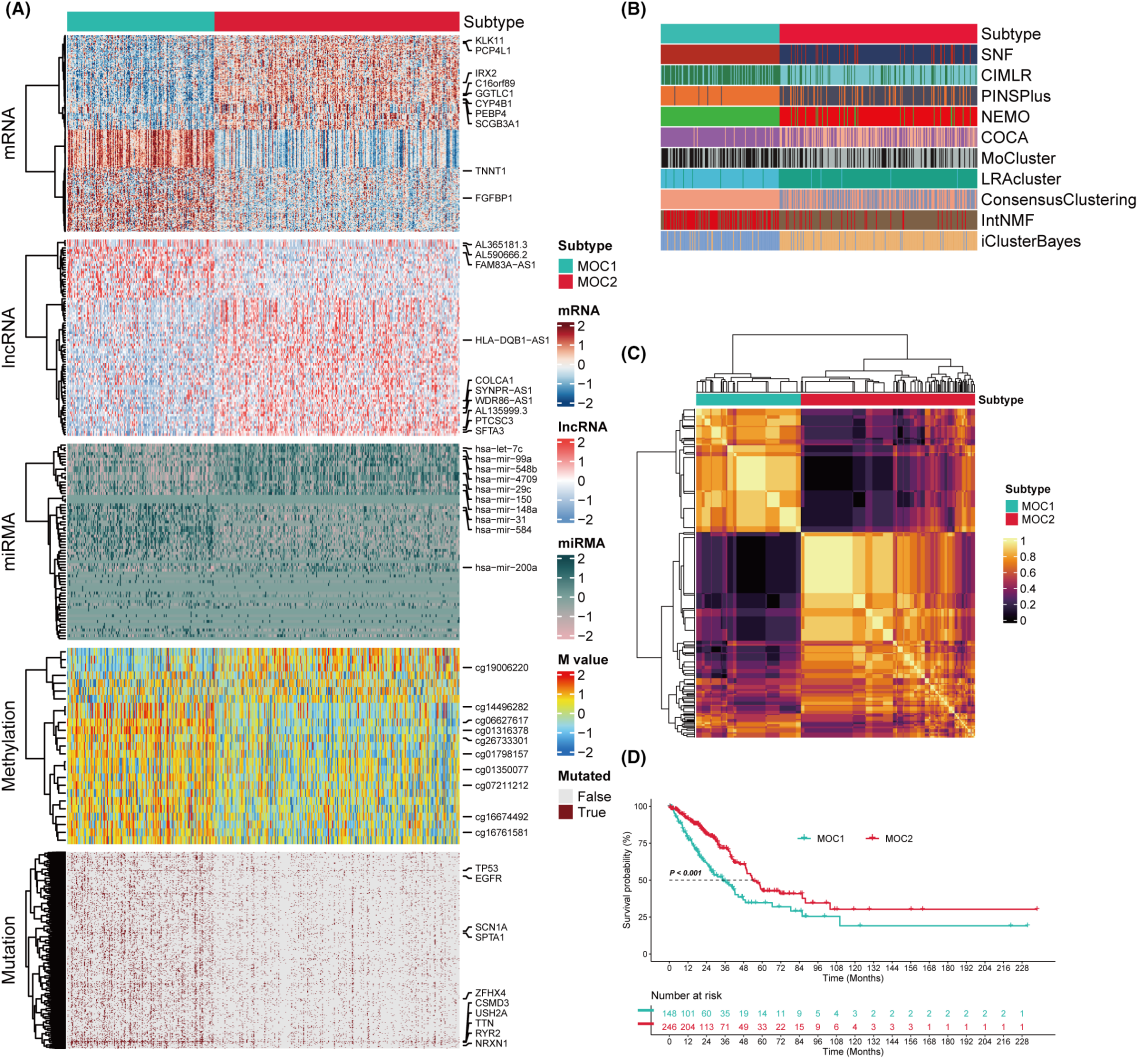
Multi‐Omics Integration and Consensus Clustering in LUAD. (A) Comprehensive heatmap of consensus clustering, encompassing mRNA, lncRNA, miRNA, DNA methylation sites and mutated genes. (B) Clustering analysis of LUAD patients utilizing ten distinct clustering algorithms. (C) Two novel types of consensus clustering matrices based on the ten clustering algorithms. (D) Differential survival outcomes between the two identified MOCs.

Following effective preprocessing of all datasets, batch effects within the data were further assessed and mitigated using PCA, confirming the elimination of batch effects (Figure S1). Independently, two subtypes were identified from ten multi‐omics integrated clustering algorithms, with the number of subtypes determined by synthesizing the CPI, Gap Statistic analysis, Silhouette Score and insights from prior studies (Figure S2 and S3). Subsequently, a consensus integration approach was employed to align the clustering results with distinct molecular expression patterns of transcripts (mRNA, lncRNA and miRNA), epigenetic methylation and somatic mutations (Figure 2A–C), clearly segregating LUAD patients into two subtypes (MOC1 and MOC2). MOC2 demonstrated a more favourable survival outcome (*p* < 0.001, Figure 2D).

**FIGURE 3:**
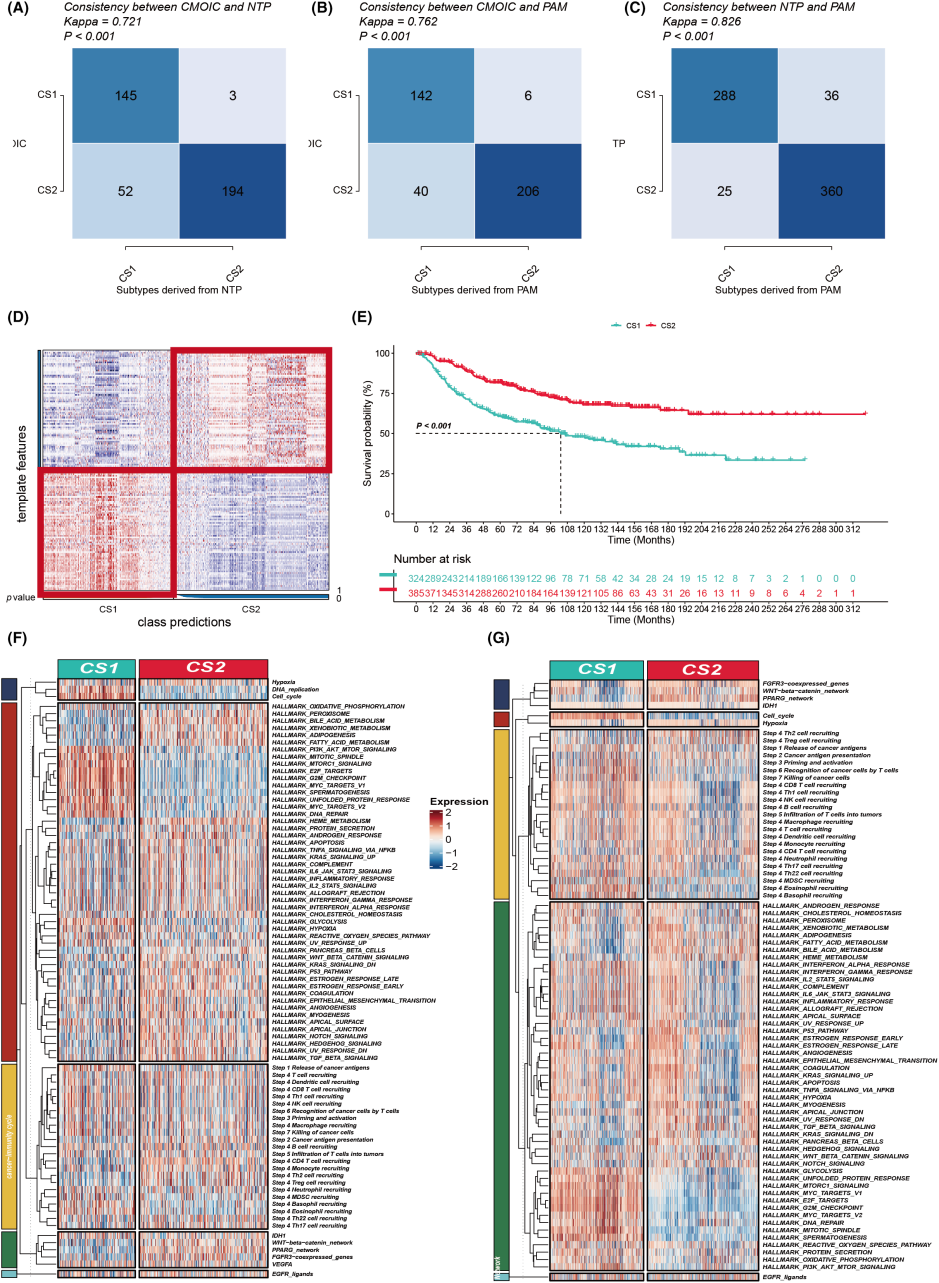
Validation and Pathway Diversity of LUAD MOCs. (A) Alignment of MOCs with Nearest Template Prediction (NTP) within the TCGA‐LUAD cohort. (B) Correlation of MOCs with Partitioning Around Medoids (PAM) clustering in the TCGA‐LUAD series. (C) Consistency between NTP and PAM classifications in the META‐LUAD collection. (D) Utilizing NTP to determine MOCs in the META‐LUAD cohort. (E) Survival outcomes associated with LUAD MOCs within the META‐LUAD cohort. (F, G) Discrepancy in pathway activities across the TCGA‐LUAD and META‐LUAD cohorts, including hallmark gene sets, cancer‐immunity cycle steps and treatment‐related signatures.

The top 100 genes specifically upregulated in each MOC were selected as classifiers and validated within an integrated external cohort to further ascertain the stability of the subtypes. Each sample within the external cohort was classified into one of the identified MOCs using Nearest Template Prediction (NTP). In alignment with this, compared to MOC1, MOC2 demonstrated better prognosis in the external LUAD cohorts (combined across six cohorts) (*p* < 0.001, Figure 3D,E). Moreover, the concordance between the MOCs algorithm and both the NTP and PAM algorithms was evaluated, showing significant consistency (*p* < 0.001; Figure 3A–C).

### 3.2 Dissecting molecular pathway enrichment in LUAD subtypes: Implications for tumour immunogenicity and therapy response

Currently, the molecular subtyping of most LUAD is classified based on gene expression levels, which may correlate with specific biological functions. Accordingly, we endeavoured to investigate the disparate molecular characteristics of these MOCs. The ssGSEA algorithm was employed to quantify the enrichment of different biological functions. When we delve into the pathway enrichment of MOC1 and MOC2, we gain a better understanding of their roles in tumour biology. The significant enrichment of DNA replication pathways in MOC1 suggests that MOC1 tumours may lean towards rapid cell proliferation and proliferation‐related biological processes. The enrichment of the cell cycle indicates that MOC1 tumour cells are more likely to be in an active proliferative state, potentially leading to a resistance to treatment. Additionally, the enrichment of E2F targets suggests that MOC1 tumours may be more susceptible to regulation by the E2F transcription factor family, which is crucial for the expression of genes involved in cell proliferation. In contrast, in MOC2, the significant enrichment of apoptosis, inflammatory response, interferon‐alpha and interferon‐gamma pathways reveals a different tumour biology profile. The enrichment of apoptosis pathways implies that MOC2 tumours may be more susceptible to apoptosis regulation, which could be a natural anticancer mechanism. The enrichment of inflammatory response pathways suggests that MOC2 tumours may be more influenced by the immune system, potentially attracting more immune cell infiltration into the tumour microenvironment. The enrichment of interferon‐alpha and interferon‐gamma pathways suggests that MOC2 tumours may be more susceptible to regulation by interferon signalling pathways, which are crucial for immune cell activation and antitumor immune responses. Furthermore, MOC2 also shows more extensive involvement in cancer‐immunity cycle pathways, including the recruitment of infiltrating T and B cells. This suggests that MOC2 tumours may be more responsive to immunotherapy, as the infiltration of immune cells may enhance their response to immune checkpoint inhibitors and CAR‐T cell therapy. In contrast, MOC1 appears to be more involved in the infiltration of MDSC cells, which may enhance the immune suppressive features of MOC1 tumours, thereby reducing sensitivity to immunotherapy (Figure 3F,G).

### 3.3 MOCM identification through advanced machine learning and clinical cohort analysis

**FIGURE 4:**
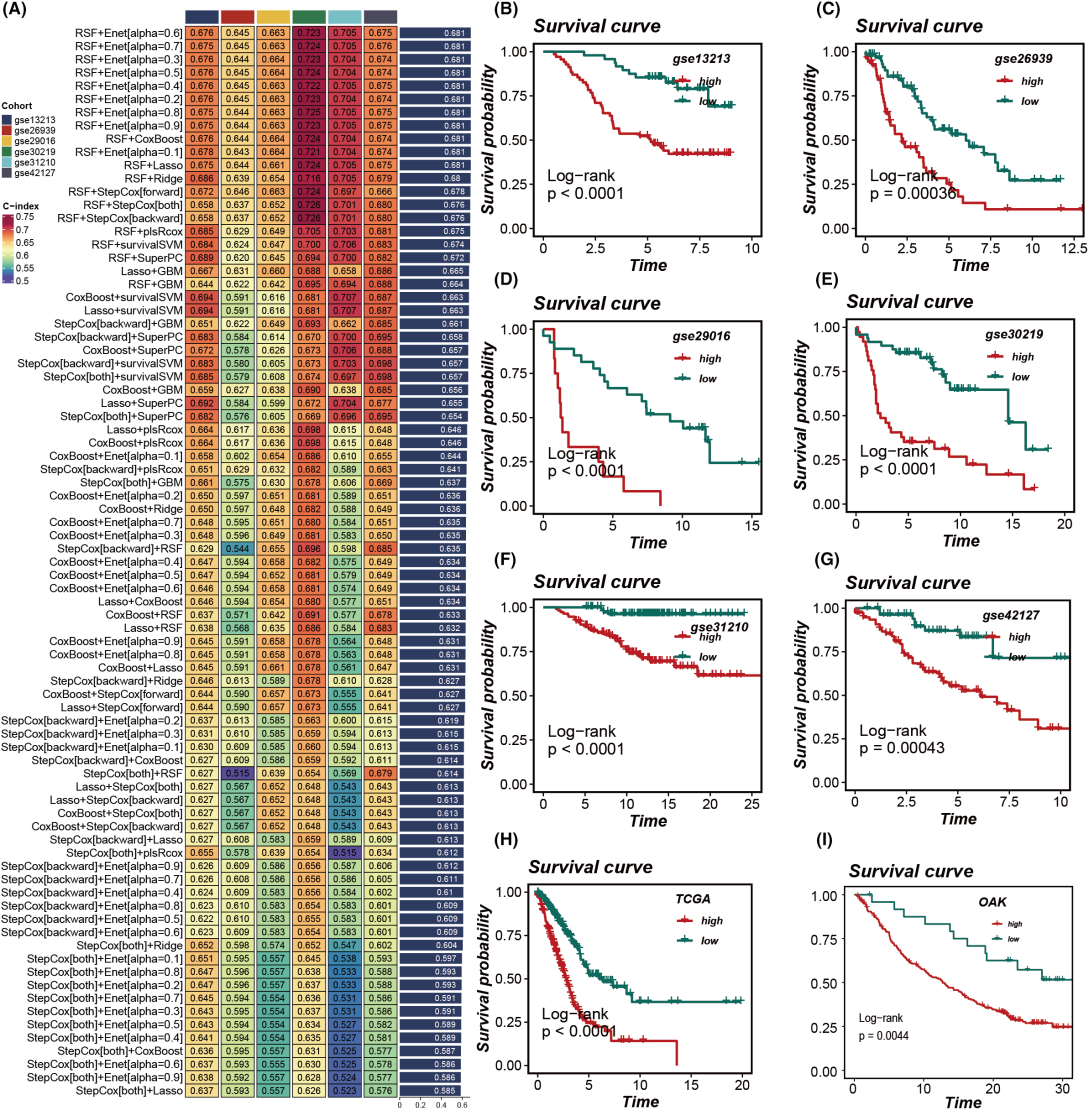
Construction of the MOCM and Its Prognostic Value. (A) A composite machine learning framework was devised, integrating various algorithmic combinations. The TCGA database served as the training cohort, while GSE13213, GSE26939, GSE29016, GSE30219, GSE31210 and GSE42127 constituted the validation cohorts for computing the concordance index (C‐index) of each model, with models ranked by the average C‐index across validation sets. (B‐H) Survival analyses comparing patients with high and low MOCM scores across six validation cohorts. (I) Expansion of the model to LUAD cohorts undergoing immunotherapy revealed that higher MOCM scores correlated with poorer survival following immunotherapy.

Further analysis was conducted on the top 100 subtype‐specific genes, where initial screening for key prognostic genes was performed using LASSO, Stepwise Cox (both forward and backward), Random Survival Forests (RSF) and CoxBoost. This was followed by an integration of five additional algorithms, constructing machine learning models through pairwise combinations, with model efficacy evaluated using the C‐index. The effectiveness of these models was evaluated using the C‐index, with the model achieving the highest average C‐index across six external cohorts deemed most optimal (Figure 4A). Ultimately, the combination of RSF with Elastic Net (Enet) [alpha = 0.6] emerged as the superior model, demonstrating a consistent impact on survival across seven datasets. Higher MOCM scores were associated with poorer survival outcomes (Figure 4B–H). Remarkably, MOCM scores calculated from our model were still able to distinguish prognoses within the OAK cohort—a randomized controlled trial focusing on immunotherapy in NSCLC, specifically extracting LUAD patients undergoing immunotherapy (Figure 4I). This indicates that MOCM can differentiate survival among LUAD patients in immunotherapy cohorts.

### 3.4 Assessing the robustness and superiority of MOCM as a prognostic signature

**FIGURE 5:**
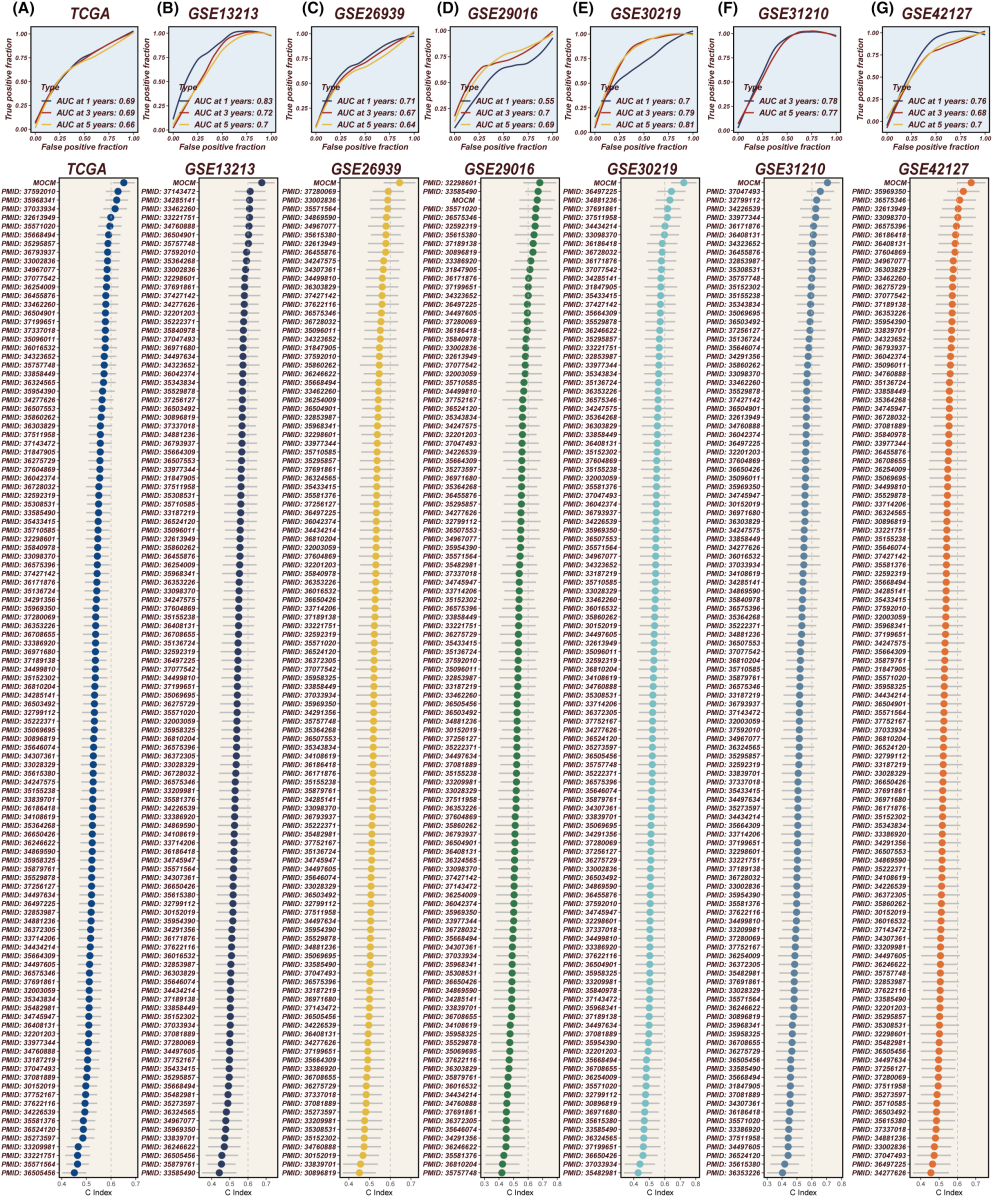
Model Evaluation. (A‐G) The ROC curves at 1–3‐5 years in TCGA, GSE13213, GSE26939, GSE29016, GSE30219, GSE31210 and GSE42127 cohorts exhibit the consistent prognostic performance of the MOCM across multiple datasets, with AUC >0.65 in all cohorts. The C‐index serves as the metric, corroborating the superior accuracy of MOCM in prognostication compared to previously published biomarkers.

With the advent of next‐generation sequencing technologies, numerous gene expression‐based prognostic markers have been extensively reported in recent years. The MOCM was evaluated across seven cohorts for its 1‐year, 3‐year and 5‐year ROC curves, demonstrating consistently high AUC values **(**Figure 5A–G **)**. To facilitate a comprehensive comparison between MOCM and other signatures, we conducted a systematic search of the literature on prognostic markers for LUAD, ultimately including 114 distinct signatures in our study. The results revealed that our constructed MOCM ranked first in six of the cohorts, underscoring its robust and superior performance (Figure 5A–G). PCA was conducted on the gene expression levels from the model across seven cohorts, demonstrating that all seven cohorts distinctly separated into two clusters (Figure S4A–G). This finding further underscores the stability and consistency of the MOCM.

### 3.5 Enhanced immune infiltration and antigen presentation in the low MOCM Group

**FIGURE 6:**
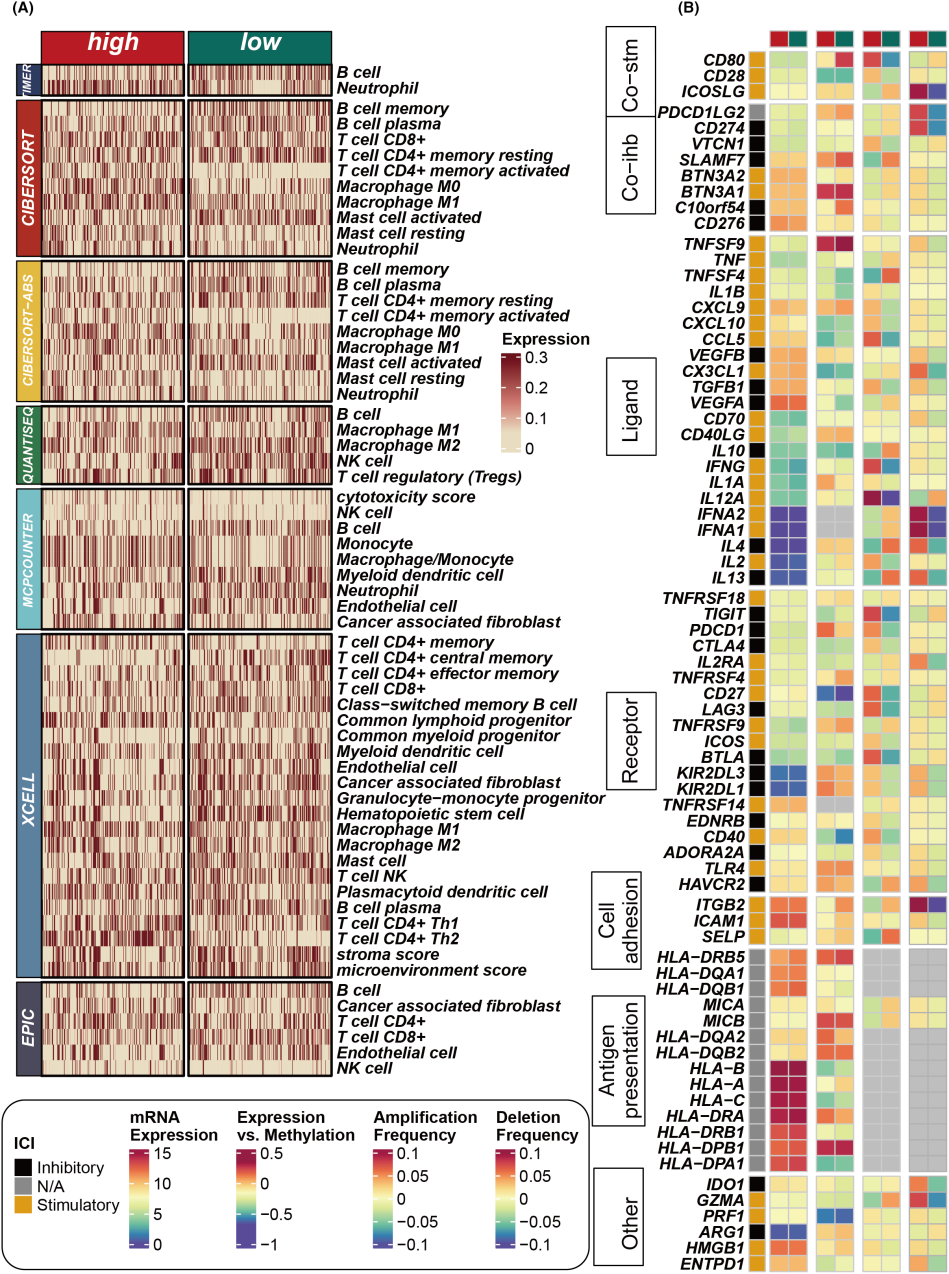
Differential Immune Infiltration. (A) Calculation of the disparity in immune cell infiltration between high and low MOCM groups utilizing seven distinct immune infiltration assessment algorithms. (B) Examination of differences in immune‐related gene expression at the mRNA, methylation and copy number variation levels between high and low MOCM groups.

Subsequently, utilizing seven different methodologies to assess immune cell infiltration, we quantitatively analysed the disparities in immune infiltration between the high and low MOCM groups. Our findings consistently revealed an enriched presence of immune cells in the low MOCM group, notably including B cells, CD4+ T cells, CD8+ T cells and natural killer (NK) cells **(**Figure 6A **)**. This pronounced infiltration suggests a more active and robust immune response within the TME of the low MOCM group. Further assessments were carried out on the expression of immune‐related genes between the high and low MOCM groups, unveiling that patients within the low MOCM group manifested elevated expressions of such genes. Specifically, expressions of HLA class molecules, including HLA‐DRB5, HLA‐DQA1 and HLA‐DQB1, were significantly higher in the low MOCM group **(**Figure 6B **)**. These molecules are pivotal in antigen presentation, a critical process for initiating and sustaining an effective immune response against tumour cells. Elevated expressions of these molecules indicate a heightened capability for antigen presentation in the low MOCM group, thereby facilitating more effective recognition and elimination of tumour cells by the immune system.

The enhanced antigen‐presenting ability within the low MOCM group likely contributes to the activation and recruitment of various immune cells into the TME. This orchestrated immune response not only aids in the direct elimination of tumour cells but also supports the establishment of an immunologically active tumour milieu, potentially inhibiting tumour progression and metastasis. Consequently, this enriched immune infiltration and heightened immune activity are indicative of a more favourable prognosis for patients within the low MOCM group. This immune landscape underscores the therapeutic potential of leveraging the immune system's natural surveillance mechanisms to improve clinical outcomes in LUAD patients, particularly those classified within the low MOCM group.

### 3.6 Evaluating the prognostic impact of model genes in pan‐cancer analysis

**FIGURE 7:**
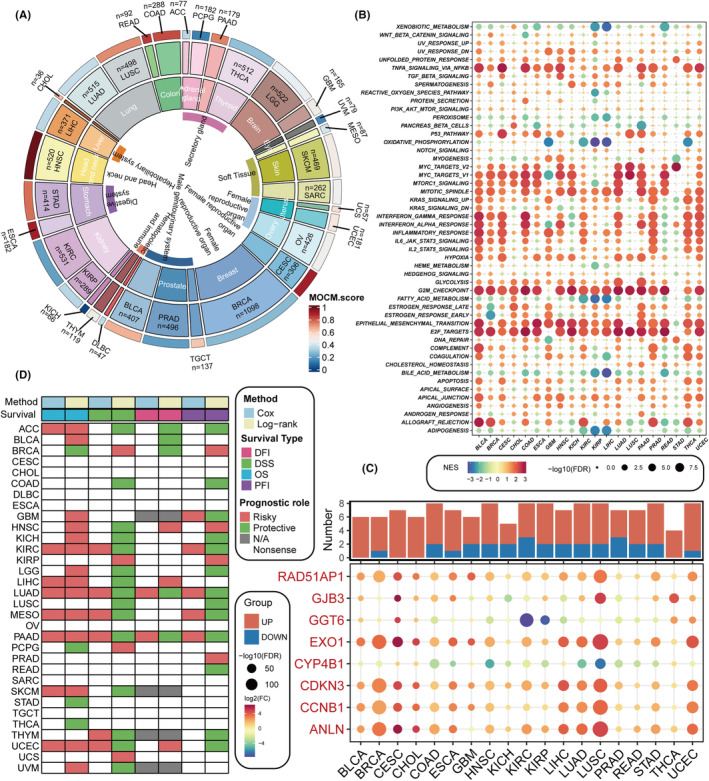
Pan‐Cancer Comparative Analysis. (A) Average level of MOCM scores across pan‐cancer scale. TCGA pan‐cancer gene expression profiles are utilized to calculate MOCM scores for different tumour types, displaying the average MOCM scores of patients with various cancer types. (B) Enrichment analysis for hallmark pathways compares tumours with high versus low MOCM scores, with enrichment quantified by the GSEA‐derived Normalized Enrichment Score (NES). (C) The histogram in the upper panel delineates the count of genes exhibiting significant differential expression, whereas the heatmap depicts fold change and false discovery rate (FDR) for model genes across diverse cancers. Genes significantly upregulated are highlighted in red; downregulated are in green. (D) A panoramic view of the correlation between GJB3 expression and patient outcomes, including overall survival (OS), disease‐specific survival (DSS), disease‐free interval (DFI) and progression‐free interval (PFI), utilizing univariate Cox regression and Kaplan–Meier analyses. Red denotes GJB3 as a prognostic risk factor in cancer; green indicates a protective factor. Only *p* < 0.05 are reported.

Subsequently, we explored the potential survival impact of eight model genes within the MOCM, revealing that patients with high expression levels of ANLN, CCNB1, CDKN3, EXO1, GJB3 and RAD51AP1 exhibited poorer overall survival, while GGT6 and CYP4B1 acted as protective genes, with their high expression associated with better survival outcomes (Figure S5A–H). We then extended the MOCM scores to pan‐cancer settings, observing the expression patterns of MOCM scores across various cancers (Figure 7A). Through GSEA of the transcriptomes between high and low MOCM groups, we investigated the differences in tumour hallmark gene sets. Cell cycle‐related pathways, such as G2M_CHECKPOINT, E2F_TARGETS, MYC_TARGETS_V1 and MYC_TARGETS_V2, were found to be enriched in the high MOCM group (Figure 7B). Further analysis of the model genes within MOCM across pan‐cancer settings revealed that ANLN, CCNB1, CDKN3, EXO1, GJB3 and RAD51AP1 were predominantly overexpressed in tumour tissues, while GGT6 and CYP4B1 were highly expressed in normal tissues (Figure 7C). Further analysis indicated a significant positive correlation between ANLN, CCNB1, CDKN3, EXO1, GJB3, RAD51AP1 and MOCM scores, with GGT6 and CYP4B1 showing a significant negative correlation (Figure S6A–H). Upon reviewing the literature, we noted that research on GJB3 in tumours is limited; thus, we delved deeper into the prognostic role of GJB3 across pan‐cancer settings, finding it to act predominantly as a risk factor in most tumours (Figure 7D).

### 3.7 Impact of GJB3 knockdown on proliferation, migration and invasion in LUAD cells

**FIGURE 8:**
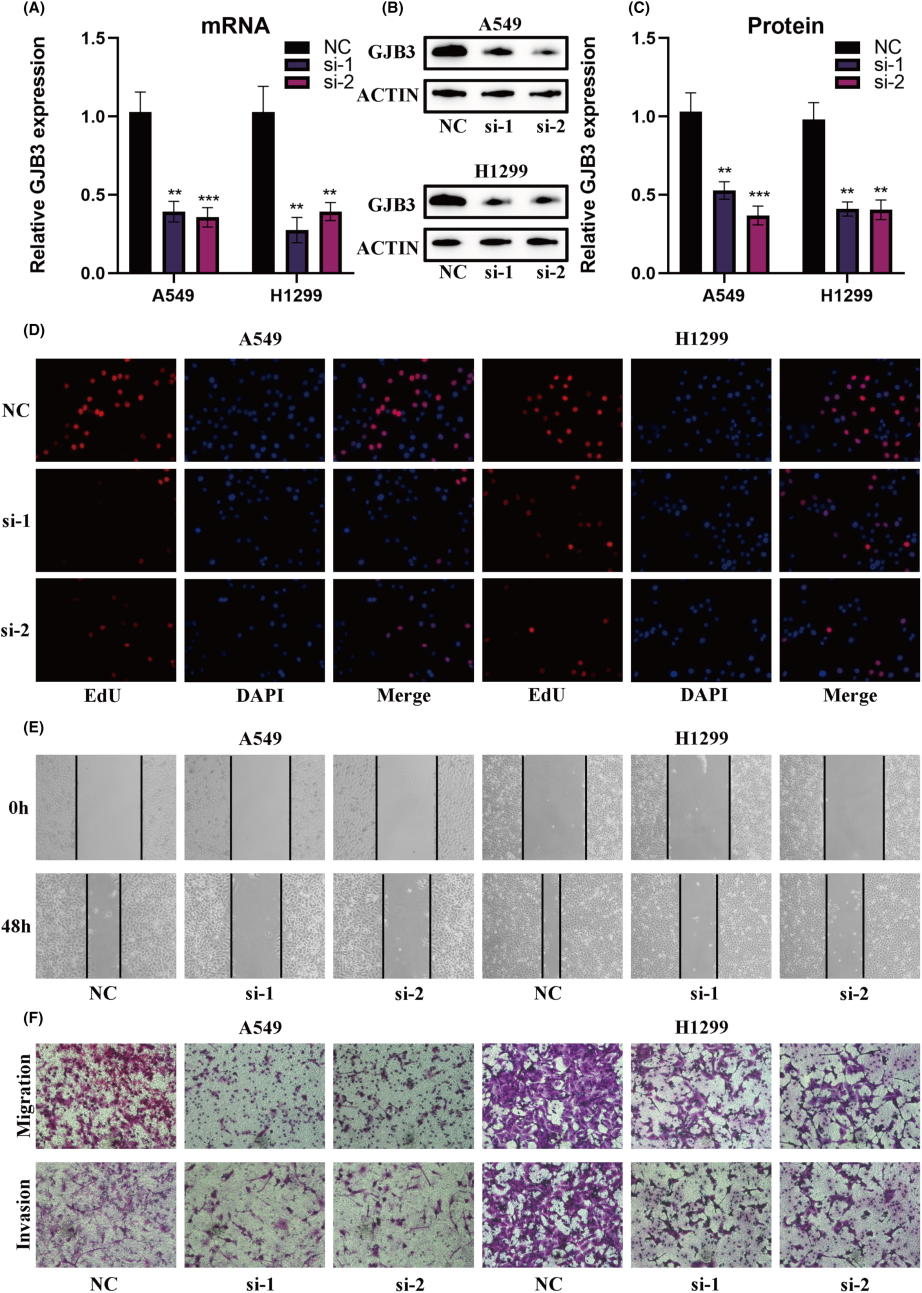
GJB3's Impact on LUAD Cell Proliferation. (A–C) Verification of targeted GJB3 reduction in A549 and H1299 LUAD cell lines via siRNA‐mediated knockdown. (D) Examination of the repercussions of GJB3 knockdown on LUAD cell proliferation rates through 5‐ethynyl‐2′‐deoxyuridine (EdU) incorporation assays, revealing a significant decrease in cellular DNA synthesis activity. (E) Analysis of the migratory capacity of LUAD cells post‐GJB3 knockdown with wound healing assays, depicting delayed migratory response. (F) Investigation of the inhibitory effects on cell invasion and migration upon GJB3 knockdown utilizing Transwell assays, highlighting GJB3's essential role in the invasive and motility attributes of LUAD cells. Notations: **p < 0.01; ***p < 0.001.

To elucidate the critical involvement of Gap Junction Beta‐3 (GJB3) in LUAD, we manipulated GJB3 RNA and protein levels in A549 and H1299 cell lines via siRNA‐mediated downregulation (Figure 8A–C). Subsequently, cellular proliferation impacts due to GJB3 knockdown were evaluated using 5‐ethynyl‐2′‐deoxyuridine (EdU) incorporation assays. The results from these assays demonstrated a significant reduction in the proliferative capacity of LUAD cells following GJB3 knockdown (Figure 8D, Figure S7A). Additional analyses were performed to assess the impact of GJB3 suppression on the migratory and invasive abilities of LUAD cells. Quantitative data from wound healing assays showed that GJB3 knockdown markedly impeded wound closure rates, suggesting a substantial decrease in cell migration (Figure 8E, Figure S7B). Furthermore, through Transwell migration and invasion assays, a notable decline in the invasive and migratory prowess of LUAD cells post‐GJB3 knockdown was observed, supporting the notion of GJB3's pivotal role in LUAD cells' metastatic characteristics (Figure 8F, Figure S7C,D). Subsequent experiments revealed that GJB3 knockdown significantly increased apoptosis in LUAD cells (Figure S8A,B, Figure S7E). Altogether, these findings shed light on GJB3's essential role in augmenting LUAD cell proliferation, migration and invasion, thereby proposing GJB3 as a viable target in the therapeutic landscape of LUAD.

## 4 DISCUSSION

Gene expression is meticulously regulated through diverse genetic and epigenetic mechanisms, including methylation, mutations and histone modifications.^27^, ^28^ Thus, an extensive analysis of multi‐omics patient data is pivotal for delving into disease‐specific regulatory mechanisms.^29^, ^30^ However, thus far, research has largely been confined to individual omics studies,^31^ and the selection of omics clustering methods is greatly influenced by personal preferences, which exacerbates the limitations of specific methodologies as their application broadens. Our study aims to address this issue by integrating ten cutting‐edge clustering algorithms to identify two prognostically distinct subtypes, offering significant implications for the precise stratification and treatment of LUAD patients, with the novelty of these subtypes being confirmed as stable across several cohorts.

Machine learning algorithms serve as a potent tool for analysing multi‐omics data.^32^ To discern the molecular characteristics distinguishing different prognostic subtypes and enhance clinical applicability, our research incorporated seven multi‐centre LUAD cohorts, designating TCGA as the training cohort and six additional datasets as validation cohorts. We then employed a combination of ten machine learning algorithms to select the optimal MOCM, aiming to surmount the biases inherent in algorithm selection. In the current landscape where artificial intelligence and vast biological datasets deeply intersect, overfitting presents a critical concern in model construction.^33^ with models performing well in training sets but facing challenges in generalization across validation cohorts. To circumvent the pitfalls of overfitting in the training cohort, we used the average C‐index across multiple validation cohorts as a benchmark for model ranking. Our findings revealed that training with RSF and Enet [alpha = 0.6] yielded superior performance in the training set but struggled with generalization across validation sets. Following this rationale, we observed that the carefully curated MOCM exhibited robust prognostic value across all cohorts, outperforming other previously published signatures.

Subsequently, we examined the differences in immune‐related characteristics between high and low MOCM groups. It was observed that in the high MOCM group, there was a decrease in various immune cell infiltrations, often associated with a ‘cold’ tumour phenotype.^34^ Conversely, the low MOCM group exhibited rich immune cell infiltration and increased expression of MHC class molecules, suggesting a potent anti‐tumour immunity,^35^ which was corroborated by survival analyses indicating a better prognosis, a finding validated in the OAK immunotherapy cohort. Additionally, among MOCM model genes, GJB3 has been understudied in LUAD, with a significant positive correlation between GJB3 and MOCM (cor = 0.77, *p* < 0.01). Our experiments confirmed its role as an oncogene, enhancing the proliferative, invasive and migratory capacities of LUAD cells, potentially marking it as a critical target for LUAD treatment.

Our study presents several distinct aspects compared to earlier research. Initially, acknowledging the pronounced heterogeneity of LUAD, we conducted a focused analysis within LUAD to enable more precise patient stratification and treatment. Second, we integrated multi‐omics information across all five dimensions in LUAD, employing a comprehensive set of ten clustering algorithms to maximize the informational content of each omics dimension while minimizing the impact of clustering method selection bias on our analysis. Third, our model was selected based on multiple cohorts, enhancing its stability and prognostic value. Fourth, by systematically integrating data from seven multi‐centre cohorts and utilizing ten widely used machine learning algorithms, we identified the model with the best average C‐index performance to establish MOCM, aiming to minimize the potential impact of overfitting on our research outcomes. Fifth, we validated the key model gene GJB3 as an oncogene potentially influencing LUAD progression and serving as a potential therapeutic target. Nevertheless, we acknowledge certain limitations in our research. For instance, the specific mechanisms by which GJB3 acts as an oncogene warrant further investigation. Moreover, the clinical utility of MOCM should be more extensively validated in larger, prospective multi‐centre cohorts.

## AUTHOR CONTRIBUTIONS

**Haoran Lin:** Methodology (equal); writing – review and editing (equal). **Xiao Zhang:** Formal analysis (equal); writing – original draft (equal). **Yanlong Feng:** Investigation (equal); methodology (equal); software (equal). **Zetian Gong:** Data curation (equal); validation (equal). **Jun Li:** Project administration (equal); writing – review and editing (equal). **Wei Wang:** Conceptualization (equal); supervision (equal); writing – review and editing (equal). **Jun Fan:** Conceptualization (equal); supervision (equal); writing – original draft (equal).

## FUNDING INFORMATION

None.

## CONFLICT OF INTEREST STATEMENT

It is hereby declared by the authors that the research was carried out without the presence of any potential conflict of interest arising from commercial or financial relationships.

## Supporting information


Figure S1.

Figure S2.

Figure S3.

Figure S4.

Figure S5.

Figure S6.

Figure S7.

Figure S8.

Table S1.

## Data Availability

The datasets analysed in the current study are available in the TCGA repository (http://cancergenome.nih.gov/), and GEO (https://www. ncbi.nlm.nih.gov/geo/).
