# Supplementary figures and images for: Advancing lung adenocarcinoma prognosis and immunotherapy prediction with a multi‐omics consensus machine learning approach
Source: J Cell Mol Med. 2024 Jul 3;28(13):e18520. doi: 10.1111/jcmm.18520 (PMC11221067; doi:10.1111/jcmm.18520)

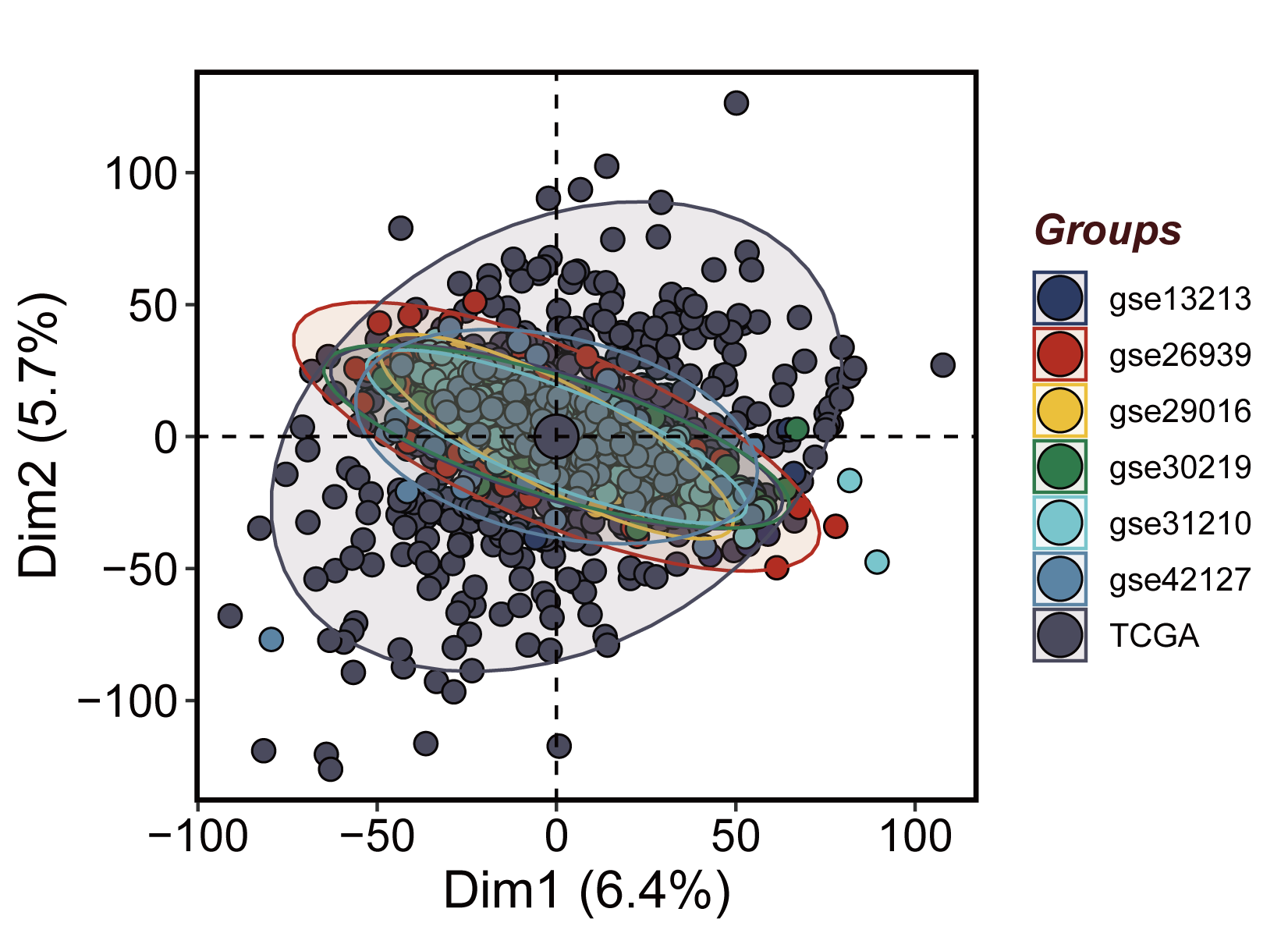

Supplement: Supplementary file 1 — Figure S1. [file JCMM-28-e18520-s005.tif]

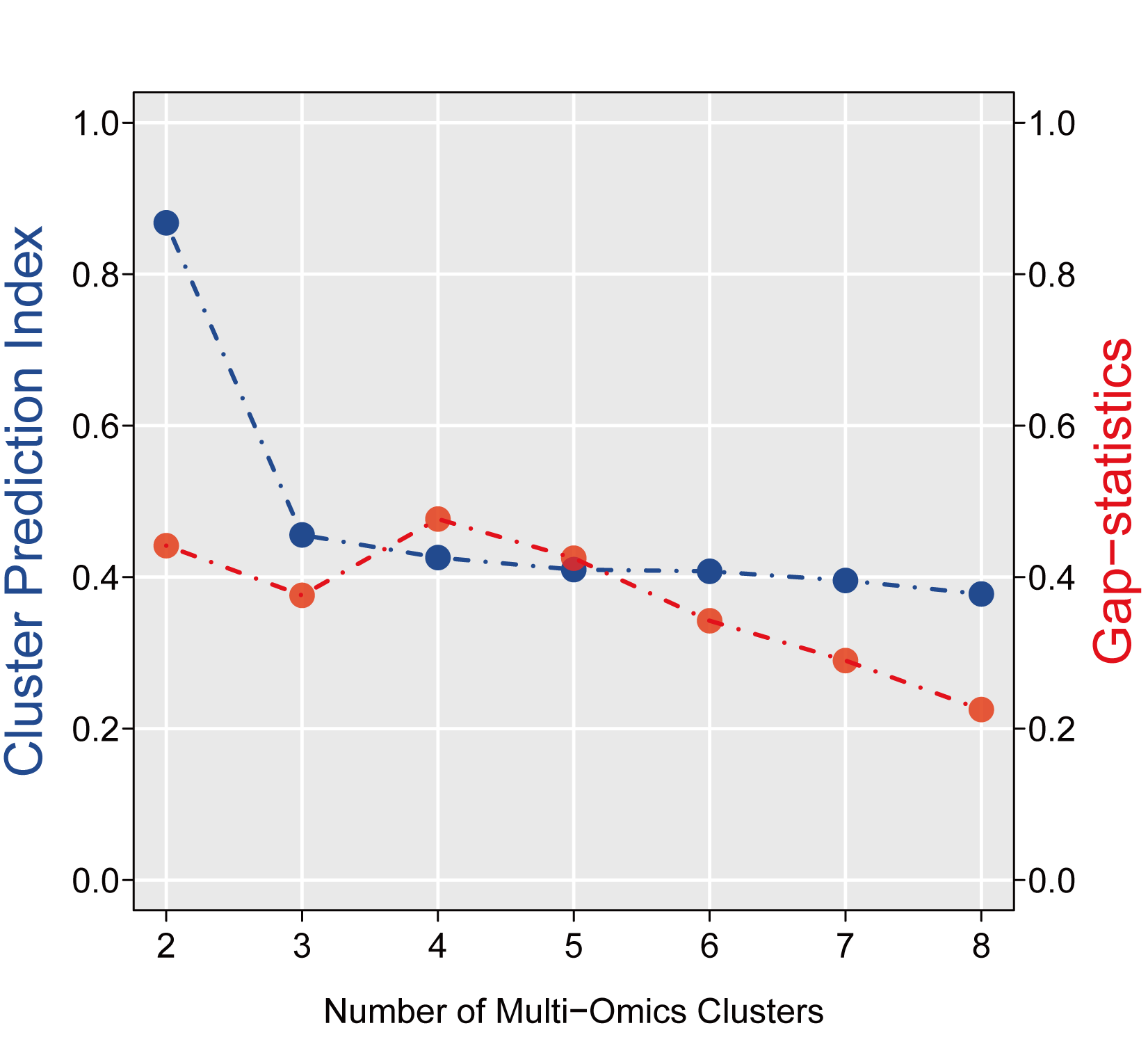

Supplement: Supplementary file 2 — Figure S2. [file JCMM-28-e18520-s003.tif]

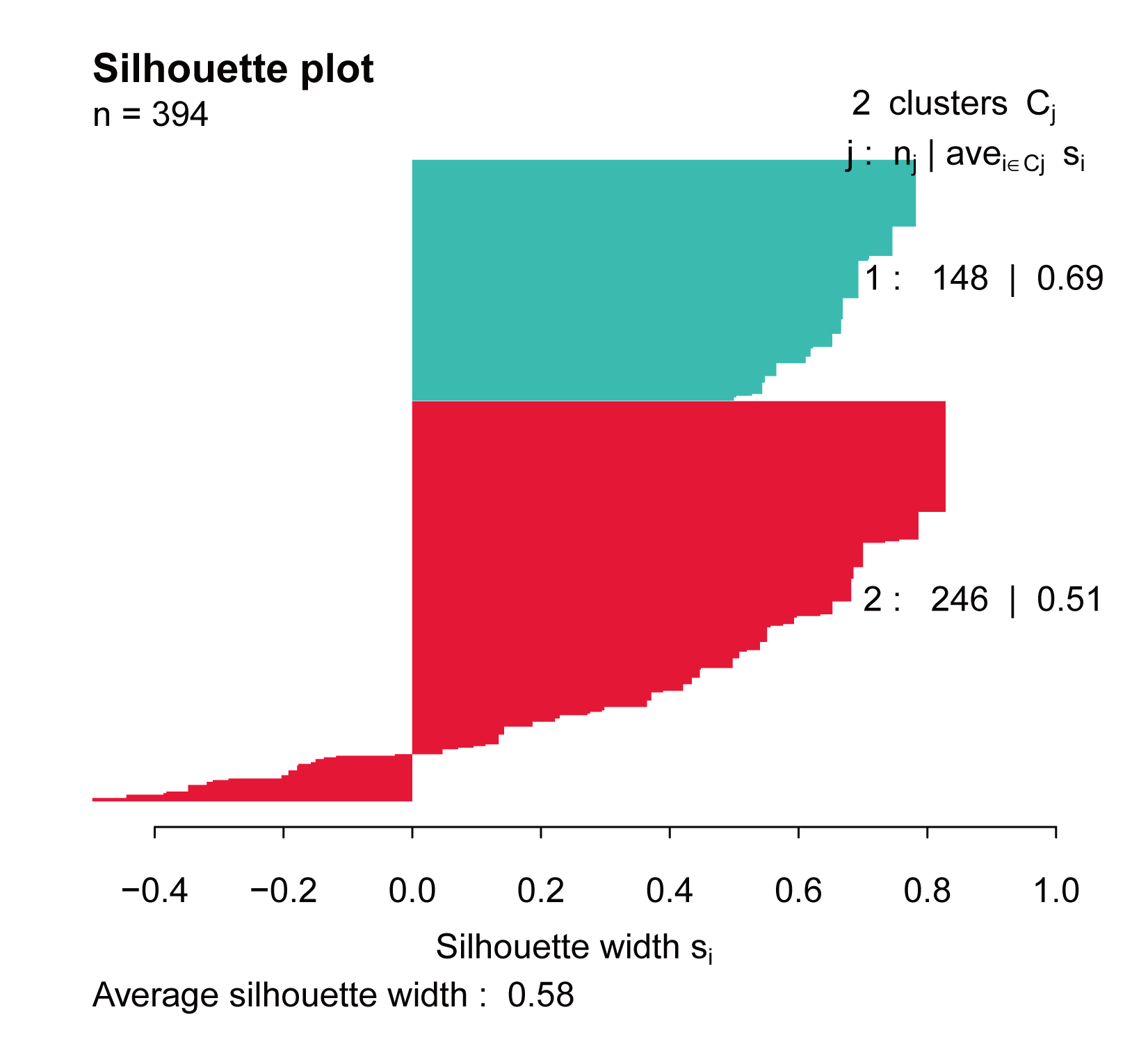

Supplement: Supplementary file 3 — Figure S3. [file JCMM-28-e18520-s006.tif]

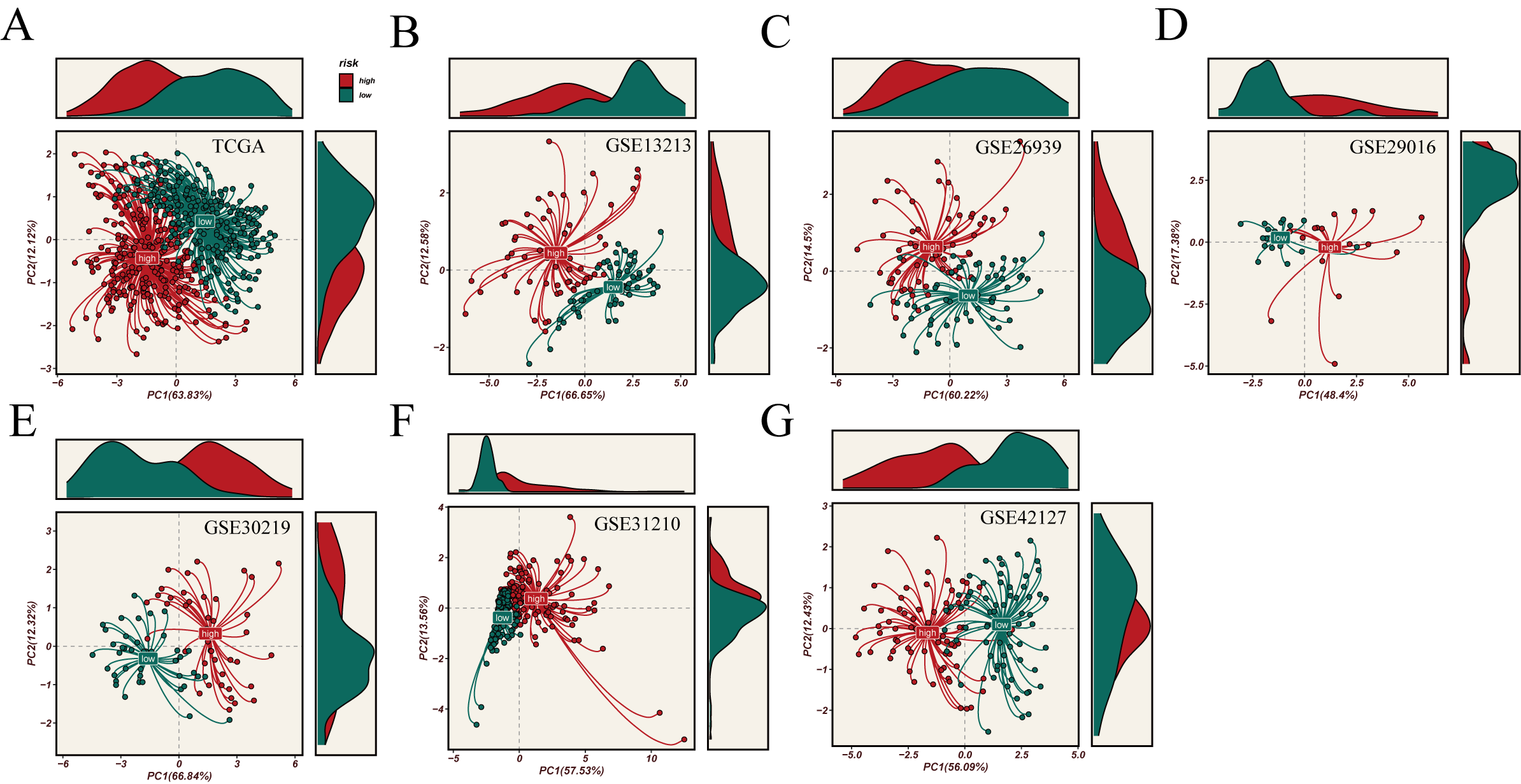

Supplement: Supplementary file 4 — Figure S4. [file JCMM-28-e18520-s007.tif]

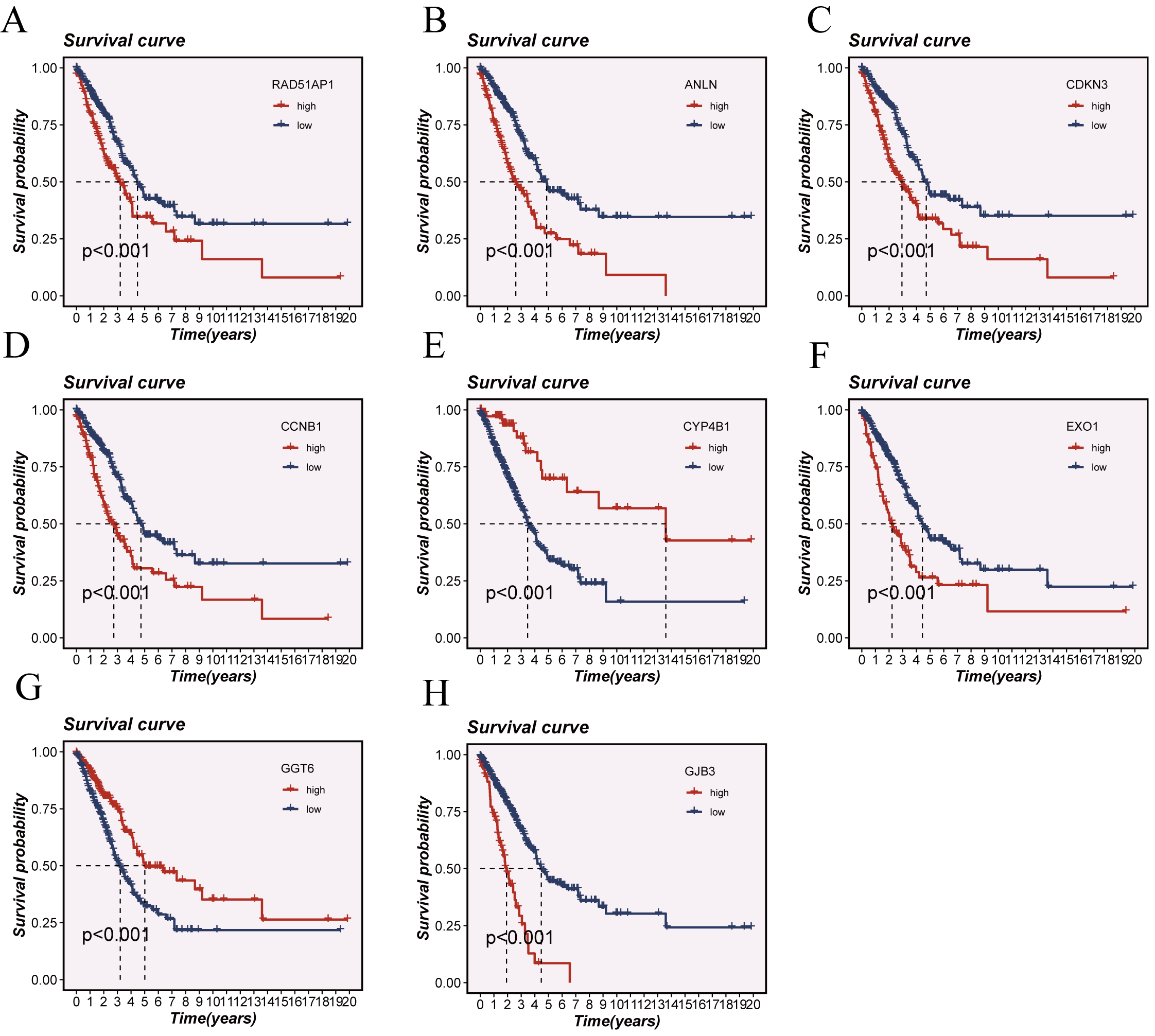

Supplement: Supplementary file 5 — Figure S5. [file JCMM-28-e18520-s009.tif]

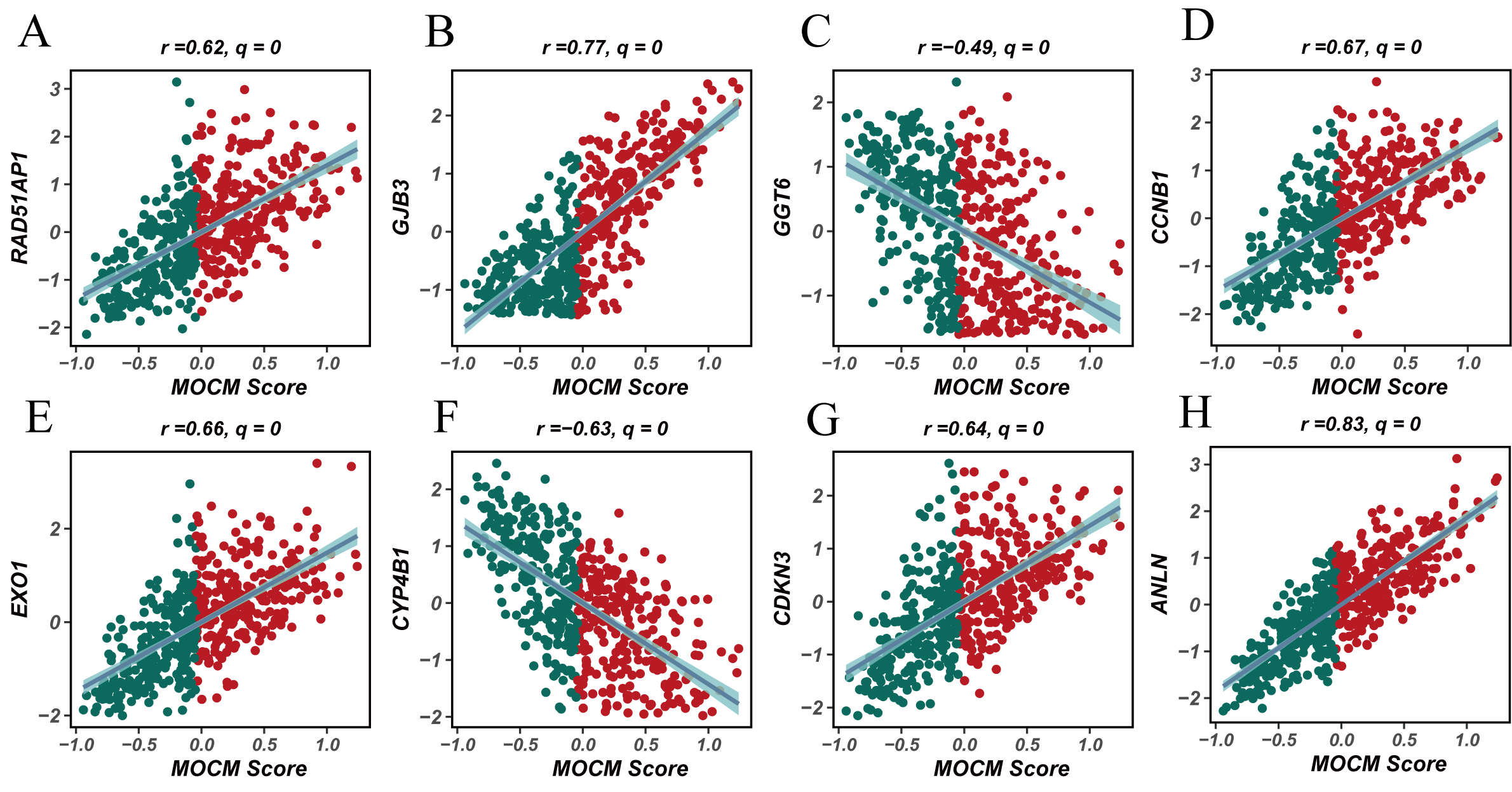

Supplement: Supplementary file 6 — Figure S6. [file JCMM-28-e18520-s004.tif]

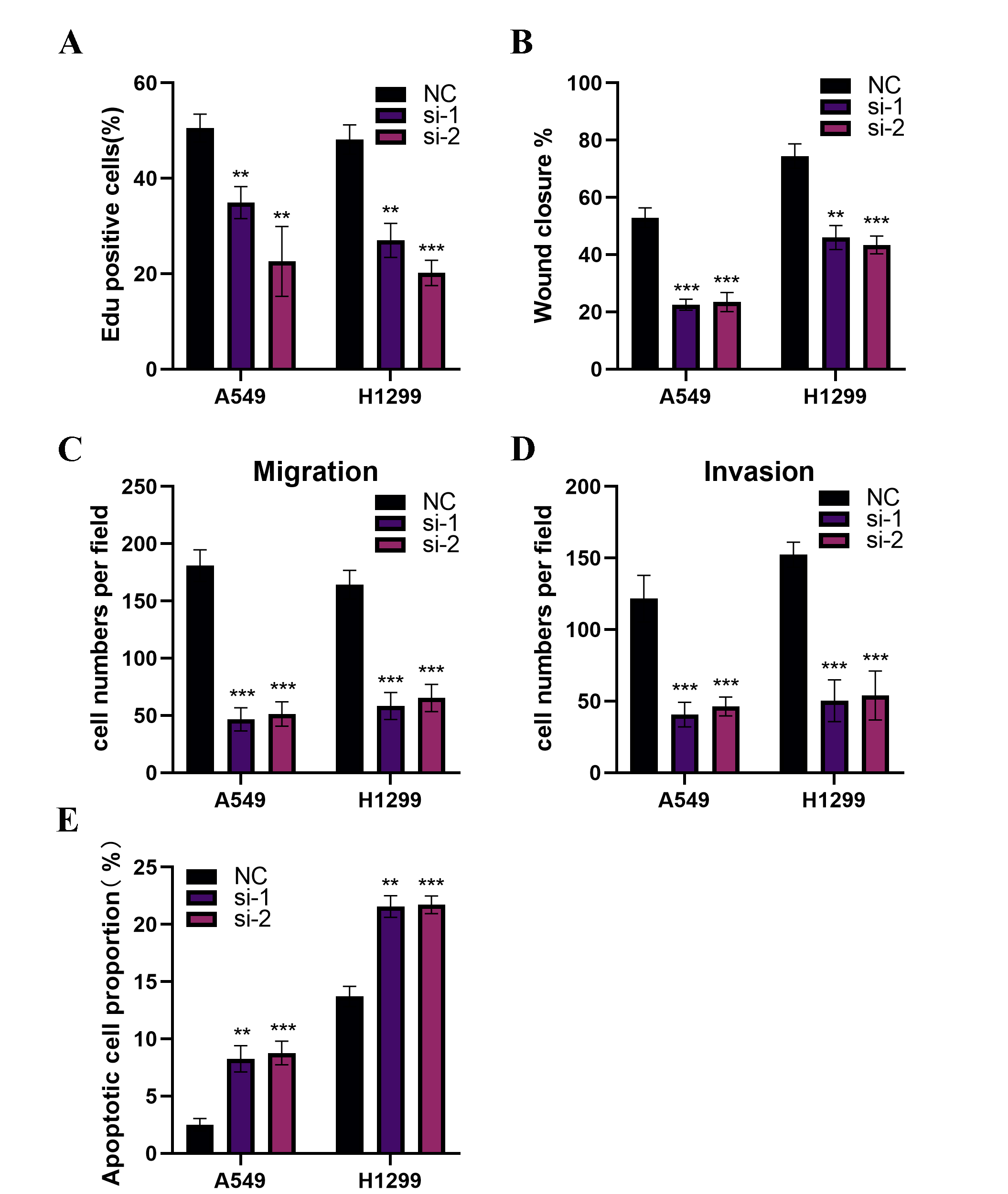

Supplement: Supplementary file 7 — Figure S7. [file JCMM-28-e18520-s001.tif]

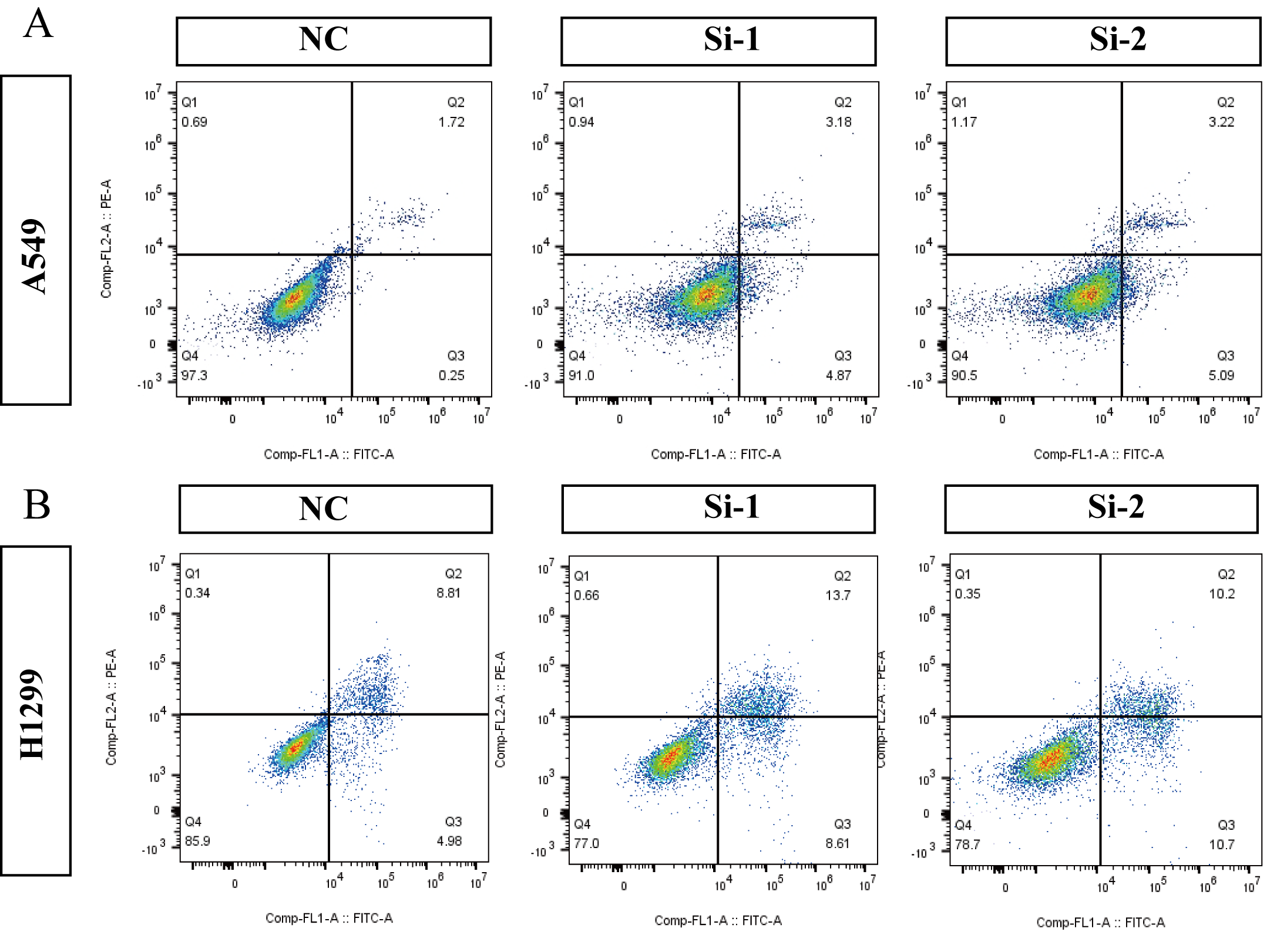

Supplement: Supplementary file 8 — Figure S8. [file JCMM-28-e18520-s008.tif]
